# Tracheoarterial fistula in a patient with amyotrophic lateral sclerosis successfully managed by overinflation of the tracheostomy tube cuff alone: a case report

**DOI:** 10.1186/s13256-023-03799-z

**Published:** 2023-02-25

**Authors:** Takashi Hosaka, Shintaro Furuno, Makoto Terada, Yumiko Hamano, Kenichi Komatsu, Katsuichiro Okubo, Yasuaki Koyama, Tetsu Suzuki, Hiroshi Tsuji, Akira Tamaoka, Taro Mizutani

**Affiliations:** 1 Division of Clinical Medicine, Department of Neurology, Faculty of Medicine, University of Tsukuba, Tsukuba, Ibaraki 305-8575 Japan; 2 Department of Internal Medicine, Ibaraki Western Medical Center, University of Tsukuba Hospital/Jichi Medical University Joint Ibaraki Western Regional Clinical Education Center, Chikusei, Ibaraki 308-0813 Japan; 3 Department of Internal Medicine, Ibaraki Western Medical Center, Chikusei, Ibaraki 308-0813 Japan; 4 Department of Otolaryngology, Ibaraki Western Medical Center, Chikusei, Ibaraki 308-0813 Japan; 5 Department of Emergency and Critical Care Medicine, University of Tsukuba Hospital, Ibaraki, 305-8576 Japan; 6 Department of Emergency and Critical Care Medicine, Hitachi General Hospital, Hitachi, Ibaraki 317-0077 Japan; 7 Department of Anesthesiology, Ibaraki Western Medical Center, Chikusei, Ibaraki 308-0813 Japan

**Keywords:** Tracheoarterial fistula (TAF), Amyotrophic lateral sclerosis (ALS), Overinflation of the tracheostomy tube cuff, Case report

## Abstract

**Background:**

Tracheoarterial fistula is the most devastating complication after tracheostomy, and its mortality, without definitive treatment, approaches 100%. In general, the combination of bedside emergency management, that is, overinflation of the tracheostomy tube cuff, and definitive treatment such as surgical or endovascular intervention is necessary to prevent the poor outcome. Patients with neuromuscular diseases such as amyotrophic lateral sclerosis are susceptible to tracheoarterial fistula because of long-term mechanical ventilation and muscle weakness.

**Case presentation:**

We describe a case of tracheoarterial fistula in a Japanese 39-year-old patient with amyotrophic lateral sclerosis with long-term ventilator management. The patient was clinically diagnosed with a tracheoarterial fistula because of massive bleeding following sentinel hemorrhage. The massive hemorrhage was controlled by overinflation of the tracheostomy tube cuff alone, without definitive treatment.

**Conclusions:**

This case suggests overinflation of the tracheostomy tube cuff alone plays an important role, semi-permanently, in the management of tracheoarterial fistula, especially in cases where surgical or endovascular intervention is not indicated. Clinicians taking care of patients with tracheostomy undergoing long-term mechanical ventilation should be aware that tracheoarterial fistula might occur following tracheostomy.

## Background

Tracheoarterial fistula (TAF) is a very rare complication after tracheostomy with high mortality. While the majority of TAF occurs within 3–4 weeks after tracheostomy, the minority occurs after long-term tracheostomy [1]. Patients with neuromuscular diseases, including amyotrophic lateral sclerosis (ALS), a common adult-onset motor neuron disease leading to respiratory failure due to progressive muscle weakness and atrophy [2], are susceptible to TAF because of long-term tracheostomy and muscle weakness [3]. The clinical course of TAF presents with rapid deterioration, and the prognosis of TAF is poor without immediate and appropriate treatments. Therefore, it is important to recognize early diagnostic signs of TAF, including sentinel tracheal hemorrhage, a small amount of hemoptysis, or pulsation of the tracheal cannula. In general, surgical or endovascular intervention is necessary to treat TAF [4]. Herein, we report a case of TAF in a patient with ALS with long-term tracheostomy, successfully managed by the overinflation of the tracheostomy tube cuff alone.

## Case presentation

**Fig.1:**
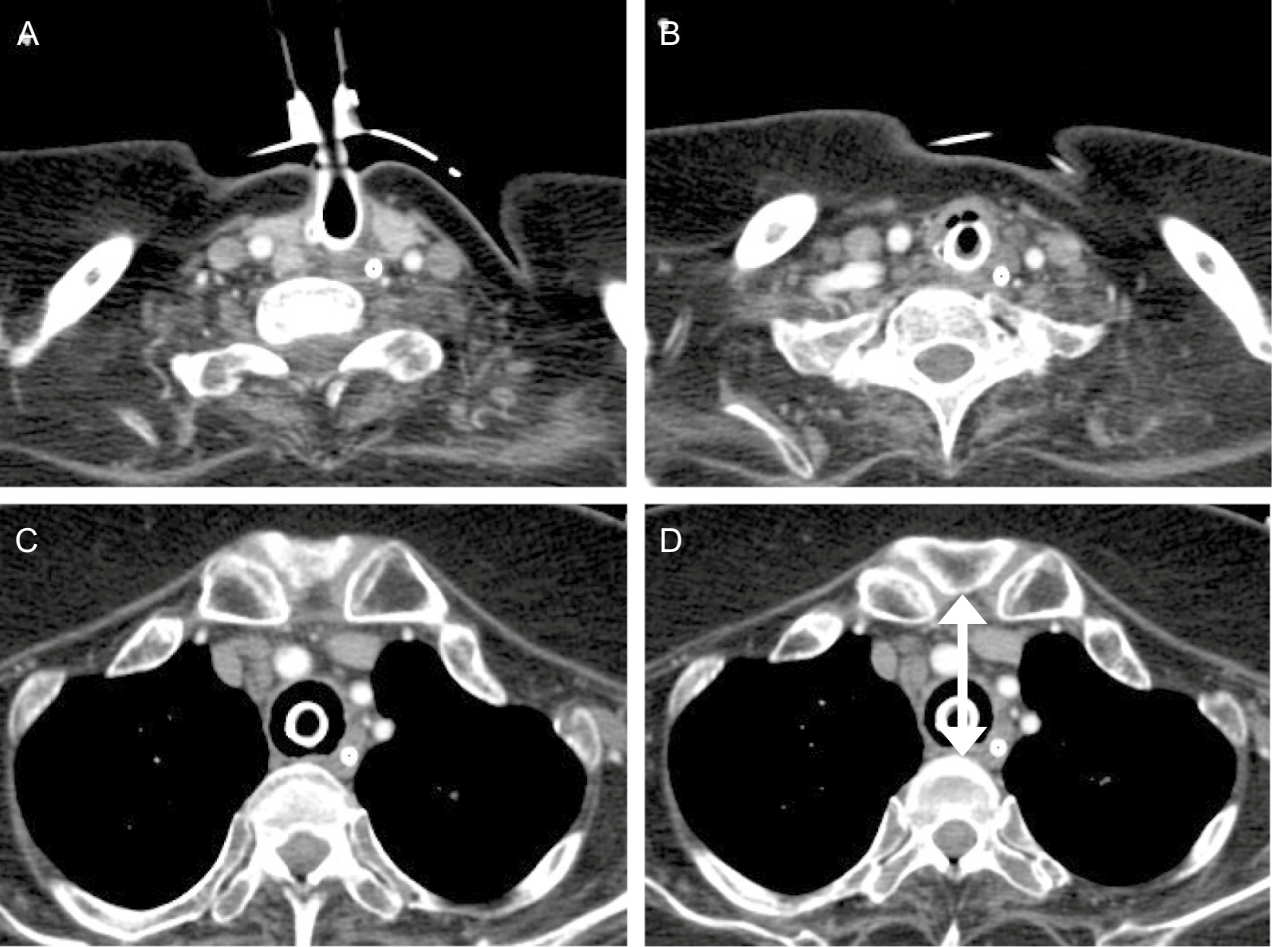
Enhanced computerized tomography (CT) on hospital day 1. **A**, **B** Tracheostomy incision was placed at the level of third tracheal ring, and no spinal and arterial deformities were observed. **C**, **D** Although the innominate artery was in close proximity of the trachea, no extravasation of contrast media was shown in enhanced CT. **D** The arrow shows the distance (46 mm) between the posterior surface of the sternum and the anterior surface of the cervical spine

A 39-year-old Japanese woman with sporadic ALS was brought to our emergency department because of massive airway hemorrhage. The patient had been mechanically ventilated for 7 years at home after tracheostomy. The patient had received neither antiplatelet nor anticoagulant agents, and her blood platelet count and coagulability were within normal limits. The hemorrhage had already stopped on admission, and the enhanced computerized tomography (CT) had not shown extravasation of contrast media (Fig. 1). Although the short distance (< 20 mm) between the posterior surface of the sternum and the anterior surface of the cervical spine may be a risk factor of TAF [5], the distance in this case was 46 mm (Fig. 1D). Bronchoscopy through tracheostoma was unable to reveal the bleeding site, signs of inflammation, or severe granulation in the stoma and the trachea.

Twenty-seven hours after initial bleeding, massive pulsatile tracheal hemorrhage occurred suddenly, causing severe desaturation. The patient was clinically diagnosed as TAF because of the massive bleeding following sentinel hemorrhage. Immediately, overinflation of the tracheostomy tube cuff was carried out to control the bleeding in the patient, and was successful. Then, the patient was transferred to a tertiary hospital to seek definitive treatment. However, it turned out that surgical or endovascular intervention was not indicated since the patient had severe comorbidities and the bleeding site was unclear. Thereafter, the patient was treated by overinflation of the tracheostomy tube cuff (33 mmHg) for 7 days, and then the cuff pressure was gradually reduced. Subsequently, tracheal hemorrhage did not occur and the patient was discharged. Since then, home care of the patient had been provided uneventfully for 20 months using an automatic tracheal tube cuff inflator.

## Discussion

In this case, although the direct evidence of TAF such as extravasation of contrast media on enhanced CT or angiographic findings was unavailable, we clinically diagnosed the patient as TAF because of the clinical course, that is, the sudden massive pulsatile tracheal bleeding following the sentinel hemorrhage. Moreover, hemorrhage due to TAF had not recurred after the initial period, which was managed by the overinflation of the tracheostomy tube cuff alone. To the best of our knowledge, this is the first report describing successful management of TAF hemorrhage by overinflation of tracheostomy tube cuff alone over months, without a need of any definitive treatment.

Since the prognosis of TAF is extremely poor, prevention is very important. The basic mechanism of tracheal injury leading to TAF is mucosal erosion and necrosis due to continuous rubbing pressure on the tracheal wall by the cuff, tip of the tracheal tube, or angulated neck of tracheostomy tube. In addition, high cuff pressure, low tracheal incision, local infection, or long-term mechanical ventilation contribute to the development of TAF [6, 7]. Patients with neuromuscular diseases are more likely to develop TAF because of long-term mechanical ventilation and muscle weakness [8–11]. Although our patient did not have common risk factors including local infection or low tracheal incision, long-term mechanical ventilation as well as the lack of cuff pressure measurement at home might be related to the development of TAF. Therefore, the tracheostomy tube cuff pressure should be measured regularly and maintained under 25 mmHg ordinarily to prevent pressure necrosis [3]. In addition, locational changes in the tip of the tracheostomy tube due to muscle weakness, which continues to progress slowly even after tracheostomy, would cause late occurrence of TAF in patients with neuromuscular diseases [9]. Therefore, periodic monitoring of the tracheostomy tube alignment seems to be desirable in patients with ALS.

Both early diagnosis and prompt management play important roles to improve the poor prognosis of TAF. For early identification of TAF, any patient having peristomal hemorrhage must be assumed to have bleeding from TAF and examined to find out the underlying causes [12, 13]. Pulsations of the tracheal cannula were reported in only 5% of the patients with TAF, whereas sentinel tracheal hemorrhage was observed in over 50% of them [6, 13]. Furthermore, although bronchoscopy and enhanced CT is sometimes helpful to detect the bleeding site, their diagnostic sensitivity is low [14]. Actually, bronchoscopy and enhanced CT in our case were unable to reveal the bleeding site. Consequently, sentinel tracheal hemorrhage would be the most available early diagnostic sign of TAF [12]. Thus, to improve the poor prognosis of TAF, if sentinel tracheal hemorrhage is observed, we should treat the patient immediately as TAF.

To avoid the fatal course of TAF, bedside emergency management with prompt diagnosis and definitive treatments are desirable. The former, which is the first step to control tracheal hemorrhage, is overinflation of the tracheostomy tube cuff. However, massive tracheal hemorrhage is not always controllable by the overinflation of the cuff alone [13]. Then, the mortality of TAF without surgical or endovascular intervention is extremely high [6, 12]. In this case, however, massive hemorrhage was controlled only by overinflation of the tube cuff and the patient survived. Tracheal tube cuff pressure over 36 mmHg causes the obstruction of blood flow in the tracheal mucosa [15]. It is widely accepted that high tracheal tube cuff pressure for long periods should be avoided concerning tracheal ischemia [16]. An experimental study using dogs with metal tubes and rubber cuffs demonstrated overinflation of tracheal tube cuff at 33 mmHg for 14 days caused tracheal stenosis later due to cartilage damage, but did not lead to apparent mucosal necrosis [17]. In our case, the overinflation of the tracheostomy cuff at a pressure of 33 mmHg continued for 7 days actually resulted in a favorable outcome, without fatal hemorrhage. Therefore, temporary overinflation at a pressure around 33 mmHg for about 7 days seemed to be a reasonable and acceptable treatment. Moreover, automatic tracheal tube cuff inflator might be an effective tool to maintain an appropriate cuff pressure.

This case report has a couple of limitations. First, regarding the diagnosis of TAF, as mentioned above, we were unable to obtain direct evidence of TAF by bronchoscopy and/or enhanced CT. A recent case series reported that, in four cases of TAF, enhanced CT was unable to demonstrate the lesion in any patient. These four patients were surgically treated after temporary overinflation of tracheostomy tube cuff and the diagnosis of TAF was confirmed [18]. Therefore, absence of positive enhanced CT findings of TAF does not preclude the diagnosis of TAF. We do believe that our patient had TAF because of the clinical findings. Second, there might be reporting bias regarding the management of TAF. It appears that TAF cases successfully treated by surgical or endovascular procedures have been actively reported [4–6, 8, 12–15, 18]. On the other hand, TAF cases with ambiguous diagnosis or unfavorable outcomes might not have been actively reported. Thus, possibly, cases of TAF managed by overinflation of tracheostomy tube cuff alone with favorable outcome exist and remain unreported due to the lack of evidence for diagnosis of TAF.

## Conclusion

This case suggests that temporary overinflation of the tracheostomy tube cuff is not only an emergency measure, but also has potential as a final treatment in patients with TAF, especially in cases where surgical or endovascular intervention is not indicated. Clinicians taking care of patients with tracheostomy undergoing long-term mechanical ventilation should be aware that TAF might occur after a long time following tracheostomy.

## Data Availability

Not applicable.

## Abbreviations

TAF: Tracheoarterial fistula
ALS: Amyotrophic lateral sclerosis
CT: Computerized tomography
